# Left ventricular myocardial work combined with stress echocardiography assessment of cardiac function in patients with Fabry disease

**DOI:** 10.1038/s41598-025-25382-w

**Published:** 2025-11-21

**Authors:** Hong Zhou, Zijie Guo, Jing Wang, Wenrui Ai, Yajing Miao, Gaojie Han, Jingchao Lu, Songyun Zhang, Hongning Yin

**Affiliations:** 1 Department of Echocardiography, The Second Hospital of Hebei Medical University, Shijiazhuang, 050000 China; 2 Department of Cardiology, Tianjin First Center Hospital, Tianjin, 300192 China; 3 Department of Cardiology, The Second Hospital of Hebei Medical University, Shijiazhuang, 050000 China; 4 Department of Endocrinology & Rare Diseases, The Second Hospital of Hebei Medical University, Shijiazhuang, 050000 China; 5 Hebei Key Laboratory of Rare Disease, Shijiazhuang, 050000 China

**Keywords:** Fabry disease, Speckle-tracking imaging, Strain imaging, Myocardial work, Exercise echocardiography, Cardiomyopathies, Cardiac hypertrophy, Cardiovascular genetics

## Abstract

Left ventricular myocardial work (LVMW) represents an innovative tool based on echocardiography designed to assess left ventricular (LV) performance in conjunction with LV pressure patterns. Although previous studies have compared differences in LVMW among patients with Fabry disease (FD), cardiac amyloidosis (CA), and hypertension at rest, there is limited research on the characteristics of LVMW in patients with FD during exercise. This study aims to explore the characteristics of LVMW at rest and during exercise in patients with FD and the value of LVMW combined with stress echocardiography for the early detection of impaired cardiac function in subclinical Fabry patients. This cross-sectional study included 54 participants, comprising 23 healthy individuals and 31 patients with FD. All participants underwent comprehensive two-dimensional echocardiography and semi-supine exercise stress echocardiography tests. At rest, individuals with FD exhibited markedly lower LV global longitudinal strain (LVGLS), LV global myocardial constructive work (LVGCW), LV global myocardial work efficiency (LVGWE), and LV global myocardial work index (LVGWI) compared to healthy controls. During exercise, LVGLS, LVGWI, LVGCW, and LV global wasted myocardial work (LVGWW) markedly increased in patients with FD and controls, while LVGWE decreased. However, across the four phases (rest, 25 W, peak, and recovery), patients with FD consistently demonstrated lower LVGLS, LVGWI, LVGWE, and LVGCW compared to controls. Moreover, the rise in LVGWI and LVGCW from the rest phase to the peak stage was markedly smaller in individuals with FD than in controls. A moderate correlation was found between LVGWI and LVGWE with LV mass index (LVMI) in individuals with FD (LVGWI: r = − 0.57, *P* < 0.05; LVGWE: r = − 0.68, *P* < 0.001). Additionally, individuals with FD with LV hypertrophy (LVH) exhibited lower LVGLS, LVGWE, and LVGCW from the rest to peak than those without LVH. Individuals with FD who had normal LVGLS at rest or those without LVH still showed markedly lower LVGWI than controls during the resting phase. Additionally, at peak exercise, LVGLS, LVGWI, and LVGCW were diminished significantly in the individuals with FD relative to the control cohort. ROC curve analysis in both resting and exercising states showed that LVGWI (rest: AUC 0.86, sensitivity 87%, specificity 74%; peak: AUC 0.94, sensitivity 71%, specificity 96%;) and LVGCW (rest: AUC 0.82, sensitivity 87%, specificity 70%; peak: AUC 0.92, sensitivity 84%, specificity 87%;) than LVGLS (resting: AUC 0.79, sensitivity 61%, specificity 87%; peak: AUC 0.88, sensitivity of 77%, and specificity of 87%) have a higher value in the diagnosis of FD. Patients with FD have markedly lower LVGWI, LVGWE, and LVGCW compared to the healthy controls, and these reductions are more prominent during exercise. Although LVGWI and LVGCW increase during exercise in patients with FD, the rate of increase is reduced, indicating impaired myocardial metabolism and energy utilization efficiency, especially in patients with FD with LVH. Additionally, LVMW combined with Stress Echocardiography allows early detection of impaired cardiac function in Fabry patients.

## Introduction

Fabry disease (FD) is an uncommon X-linked lysosomal storage disorder stemming from alterations in the *GLA* gene located at Xq22.1. These mutations result in reduced or lack of activity of the enzyme α-galactosidase A (α-GLA). The insufficiency of α-GLA results in the buildup of metabolic substrates, specifically globotriaosylceramides (Gb-3) and its derivative globotriaosylsphingosine (lyso-Gb-3), in various organs, including the skin, nerves, kidneys, and heart. This accumulation causes multi-organ damage^1^. When these substrates deposit in the heart, they contribute to myocardial fibrosis, arrhythmias, left ventricular hypertrophy (LVH), and heart failure^2^. Research has indicated that cardiac disease is the primary cause of mortality in individuals with FD^3,4^.

Echocardiography is a non-invasive and effective tool for evaluating structural and functional cardiac damage in individuals with FD^5^. Left ventricular myocardial work (LVMW) represents an innovative echocardiographic technique that integrates myocardial strain indicators with blood pressure data used to evaluate left ventricular (LV) function related to LV pressure dynamics^6^. LVMW provides a more accurate assessment of myocardial energy efficiency and function^7^ and acts as a sensitive indicator for early detection of LV dysfunction^8–10^. In addition, LVMW is an independent predictor of adverse cardiac events in patients with FD^11^. Stress echocardiography, performed under cardiac load conditions, reveals subtle changes in cardiac function that may not be evident at rest. It is vital for diagnosing and managing cardiomyopathy^12^. A decline or lack of improvement in LV ejection fraction (LVEF) and LV global longitudinal strain (LVGLS) during exercise suggests impaired LV contractile reserve, indicating a poor prognosis^13^. Stress echocardiography allows for dynamic monitoring of cardiac function, offering precise risk stratification for patients with cardiomyopathy. This method aids in evaluating treatment efficacy and provides a scientific basis for adjusting therapeutic strategies^14^.

Although a previous study has compared the differences in LVMW among patients with FD, CA, and hypertension at rest^15^, limited investigations have focused on the characteristics of LVMW in individuals with FD during exercise. Therefore, this investigation sought to explore the features of LVMW in patients with FD at rest and during exercise and the utility of combining LVMW with stress echocardiography for identifying impaired cardiac function in subclinical Fabry patients in the early stages.

## Methods

### Study population

In a single-institution observational cross-sectional investigation, 31 patients with FD (14 males and 17 females) who visited the Second Hospital of Hebei Medical University between February 2023 and April 2024 were selected. The diagnosis of FD was confirmed through α-*GLA* gene mutation analysis and α-GLA activity testing via the dried blood spot method. The patients enrolled in this study were all newly diagnosed with FD and had not yet initiated any causal treatments or cardiac medications. Individuals were excluded from the investigation if they presented with significant valvular heart issues, contraindications to exercise stress echocardiography or poor echocardiographic image quality.

A control cohort of 23 healthy individuals was also selected. These individuals had normal blood pressure, electrocardiogram results, and rest echocardiography findings, were not taking any medications, and had no history of heart disease.

All participants, including healthy individuals and patients with FD, provided informed written consent, agreeing to undergo echocardiogram assessments during rest and exercise phases, and allowed anonymized clinical data for research purposes. The research protocol was sanctioned by the Medical Research Ethics Committee of the Second Hospital of Hebei Medical University. It was executed per the ethical principles outlined in the Declaration of Helsinki.

### Echocardiography

A comprehensive echocardiographic test was conducted using a Vivid E95 ultrasound machine (GE Vingmed Ultrasound, Horten, Norway) equipped with a 1.5–4.6 MHz phased-array transducer (M5S). The modified Simpson biplane technique was employed to measure LV end-diastolic volume (EDV), LV end-systolic volume (ESV), and LVEF. The LV mass index (LVMI) was calculated utilizing the two-dimensional (2D) area-length approach, with LVH characterized as an LVMI > 88 g/m^2^ for females and 102 g/m^2^ for males.^16^ Pulsed-wave Doppler was employed to ascertain the mitral inflow early diastolic wave (E) and atrial systolic wave (A) from the apical four-chamber view. Tissue Doppler imaging was applied to acquire the early peak diastolic wave (e’) at the basal lateral wall and septum from the same apical view. The average E/e’ ratio was then calculated.

At rest, echocardiographic images were obtained and stored from parasternal short- and long-axis and apical two-chamber, four-chamber, and long-axis views. Each recording included three cardiac cycles at a frame rate of > 55 frames per second (fps). During each exercise phase (rest, 25 W, peak, and recovery), images were similarly acquired and stored from the apical two-chamber, four-chamber, and long-axis views, with each image capturing three cardiac cycles at a frame rate of > 55 fps.

LVGLS was measured using automated myocardial function imaging technology, which divides the LV wall into 17 segments and performs post-processing analysis on images from the apical two-chamber, four-chamber, and long-axis views. In cases where automated acquisition failed, manual correction of the segments was performed. The possibility of LV dysfunction increases as the absolute strain value drops below 20%^16^. A previous study has shown the non-invasive LV pressure-strain loop method correlates with invasive direct myocardial work assessments, providing insights into myocardial metabolic conditions^6^. This method integrates LV strain data with non-invasive systolic blood pressure (SBP). The peak LV systolic pressure is presumed to be equivalent to the peak arterial pressure, which is automatically documented by a blood pressure device during stress echocardiography. The curve is adjusted based on the timing of different cardiac cycle phases, defined by mitral and aortic valve events.

The following parameters were calculated:

1. LV global myocardial work index (LVGWI): The area under the LV pressure-strain loop from mitral valve closure to mitral valve opening represents the myocardium’s total work.
2. LV global myocardial constructive work (LVGCW): The myocardial work executed by the LV during systole that contributes to LV ejection, including myocardial shortening during systole and myocardial lengthening during isovolumic relaxation.
3. LV global wasted myocardial work (LVGWW): The effort that fails to contribute to LV contraction, encompassing myocardial lengthening during systole and myocardial shortening during isovolumic relaxation.
4. LV global myocardial work efficiency (LVGWE): Calculated as LVGCW/(LVGCW + LVGWW).

In this investigation, strain and strain alterations are denoted in absolute values. Data were processed offline using specialized software (GE Healthcare’s EchoPAC version 202).

### Exercise protocol

We followed the World Health Organization (WHO) 25 W protocol for a multi-stage, semi-supine bicycle exercise test. The starting workload was 25 W, increasing by 25 W every two minutes. Participants, including control individuals and individuals with FD, were encouraged to maintain a cycling speed of 55–60 rpm and continue exercising until exhaustion.

### Statistical analysis

Statistical analyses were executed utilizing GraphPad Prism 8.0.2 and IBM SPSS Statistics for Windows (version 26.0; IBM Corp., Armonk, NY, USA). Normally distributed data were denoted as mean ± standard deviation (SD), while skewed data were denoted as median with interquartile range (IQR). Categorical data were denoted as percentages. The normality of continuous data was assessed using Q-Q plots and histograms. To compare differences between cohorts, t-tests were used for normally distributed data, Mann-Whitney U tests for skewed data, and chi-square (X^2^) tests for categorical data. A linear regression model was employed to examine continuous variables. The Pearson correlation coefficient was used for normally distributed data, while Spearman’s correlation coefficient was used for skewed data. All tests were two-tailed, and *P* values < 0.05 were deemed statistically significant. The diagnostic efficacy of the model was assessed by plotting the ROC curve and calculating the area under the curve (AUC).

## Results

### Demographic and clinical characteristics

Table 1 depicts the demographic and clinical characteristics of the control cohort and individuals with FD. Compared to the control cohort, individuals with FD had a lower body mass index (BMI) and body surface area (BSA). No significant differences were noted in heart rate (HR), diastolic blood pressure (DBP), and SBP between the two cohorts.

**Table 1:** Demographic and clinical characteristics of the study population.

	Patients with FD (n = 31)	Control subjects (n = 23)	*P* value
Male (n, %)	14(45)	11(48)	0.85
Age, years	41 ± 15	41 ± 12	0.91
BSA, m^2^	1.6(1.5–1.6)	1.8(1.6–1.9)	< 0.001
BMI, kg/m^2^	21.2 ± 2.7	24.1 ± 2.2	< 0.001
SBP, mmHg	120 ± 13	123 ± 10	0.86
DBP, mmHg	75 ± 11	80 ± 7	0.07
Heart rate, bpm	70 ± 9	71 ± 9	0.78
Lyso-GL-3 increase (n, %)	30(97)	–	–
α-Gal A decrease (n, %)	25(81)	–	–
Target organ damage			
Kidney (n, %)	15(48)	–	–
Brain (n, %)	11(35)	–	–
Left ventricular hypertrophy (n, %)	19(61)	–	–
Peripheral neuropathy (n, %)	24(77)	–	–
Skin(angiokeratoma)(n, %)	8(26)	–	–

*FD* Fabry disease, *BSA* body surface area, *BMI* body mass index, *SBP* systolic blood pressure, *DBP* diastolic blood pressure.

### Resting myocardial performance

**Fig. 1:**
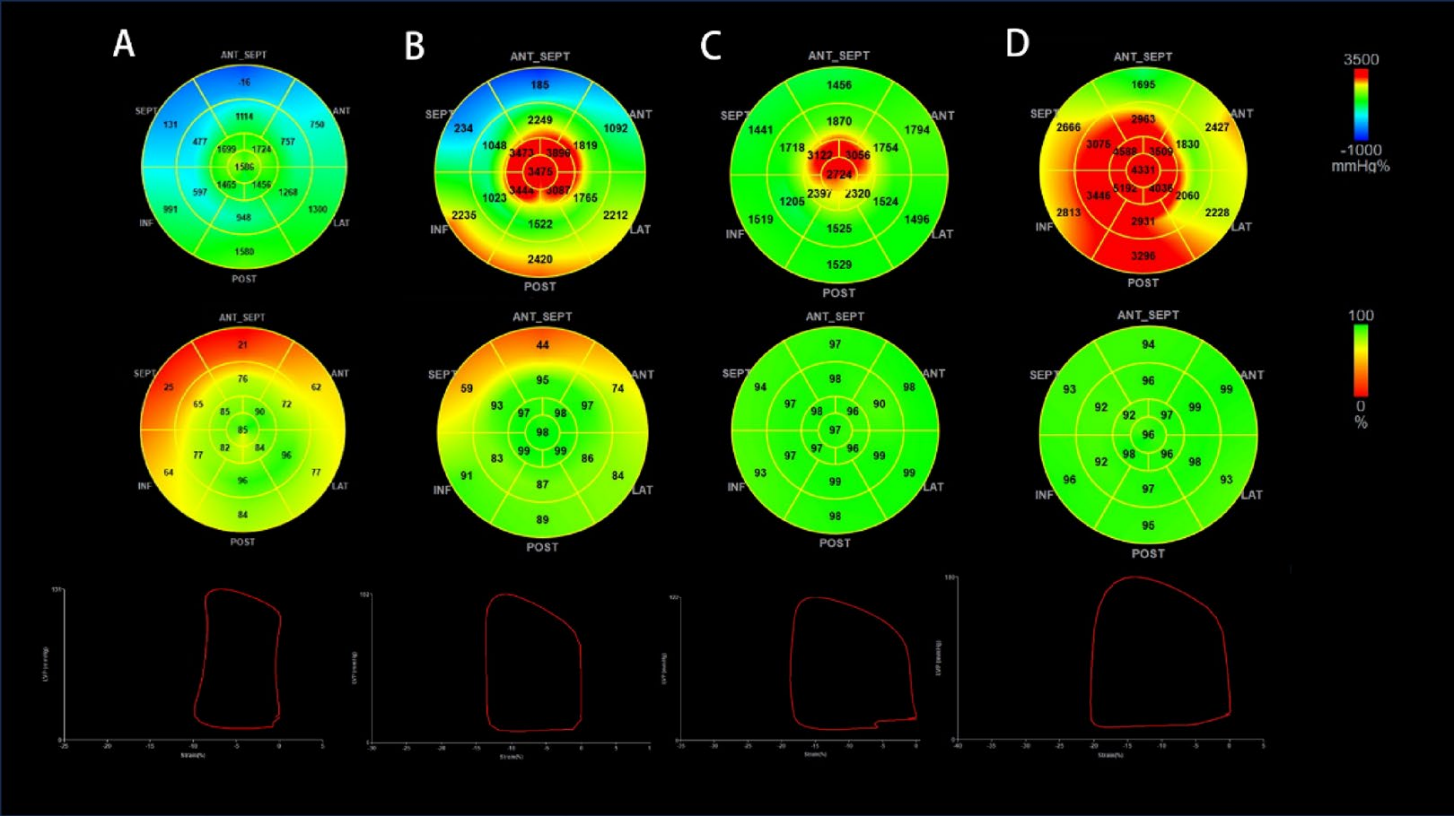
(Top panel) Seventeen-segment bull’s-eye representation of left ventricular global myocardial work index showing areas of negative work in blue, normal in green, and red indicating areas of high myocardial work; (Middle panel) 17-segment bull’s-eye representation of left ventricular global myocardial work efficiency showing areas of high efficiency coded in green and those with the least efficiency coded in red; (Bottom panel) left ventricular pressure–strain loop (PSL). Examples of individuals within: (**A**) Patients with FD at the resting phase; (**B**) Patients with FD at the peak exercise phase; (**C**) control cohort at the resting phase; (**D**) control cohort at the peak exercise phase.

**Fig. 2:**
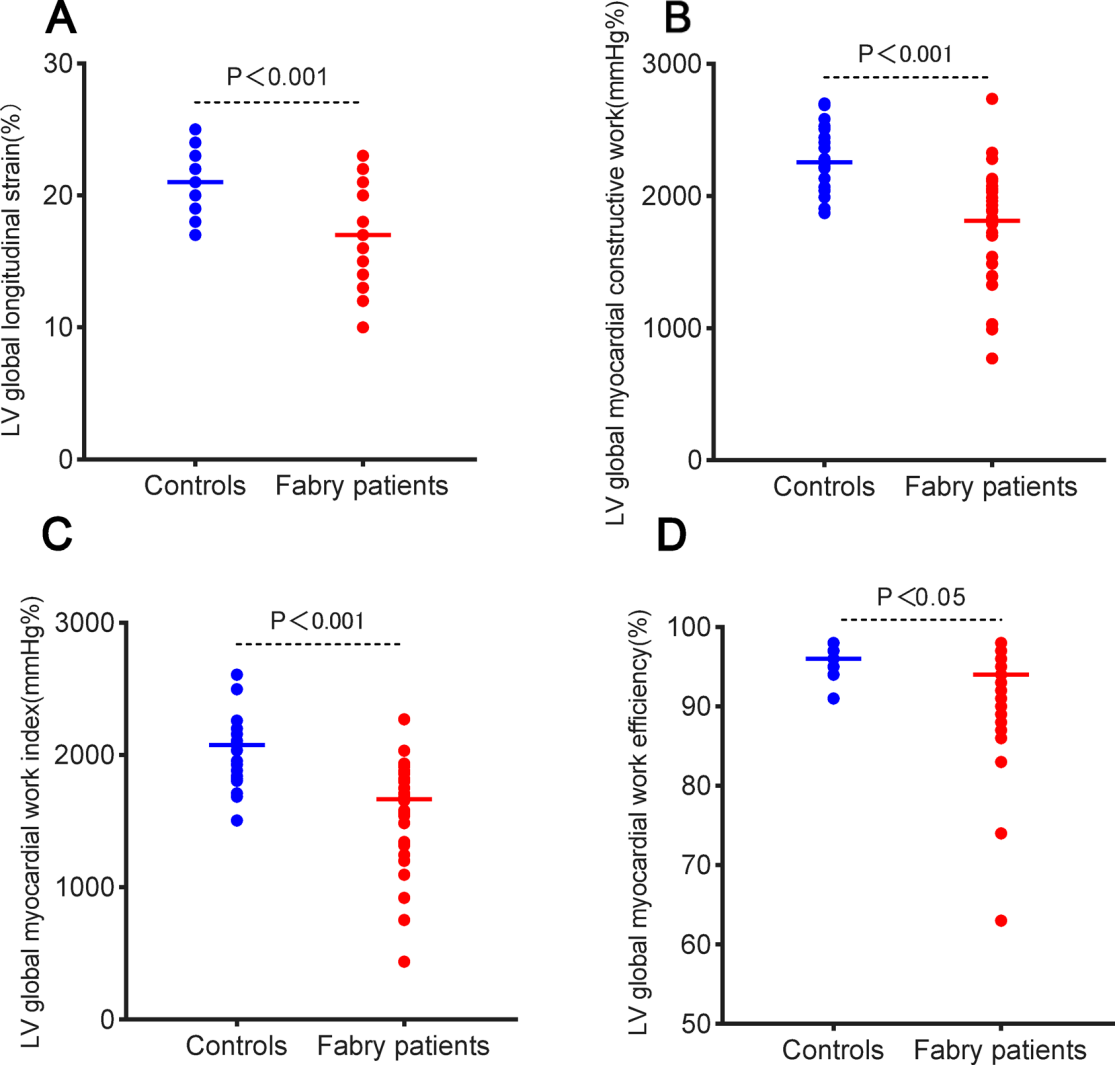
Scatterplots of (**A**) LVGLS, (**B**) LVGCW, (**C**) LVGWI, and (**D**) LVGWE in patients with FD and control subjects. *LVGLS* left ventricular global longitudinal strain, *LVGCW* left ventricular global myocardial constructive work, *LVGWI* left ventricular global myocardial work index, *LVGWE* left ventricular global myocardial work efficiency, *FD* Fabry disease.

Figure 1 demonstrates the comparative analysis of LVGWI, and LVGWE in patients with FD and the control cohort at rest and peak exercise phase. Table 2 depicts the metrics of the systolic and diastolic cardiac functions. Patients with FD showed lower LV end-diastolic diameter (LVDD) and LVEF while having increased wall thickness compared to the control cohort. Additionally, patients with FD exhibited higher LV filling pressure, as evidenced by an elevated E/e’ ratio and left atrial volume index (LAVI), suggesting worse diastolic myocardial function in individuals with FD relative to the control cohort. Advanced techniques, such as 2D speckle tracking and non-invasive LV pressure-strain loop analysis, demonstrated markedly reduced LVGLS, LVGWI, LVGWE, and LVGCW in individuals with FD relative to controls (Fig. 2). Nevertheless, no substantial difference in LVGWW between the two cohorts (*P* = 0.09).

**Table 2:** Resting echocardiography.

	Patients with FD (n = 31)	Control subjects (n = 23)	*P* value
LVDD, mm	42 ± 5	45 ± 4	< 0.05
IVS, mm	12(8–18)	9(8–9)	< 0.001
LVPW, mm	12(8–14)	9(8–9)	< 0.05
LVMI, g/m^2^	111 ± 36	71 ± 14	< 0.001
Systolic function parameters			
LVEF, %	61(57–62)	64(61–66)	< 0.05
LVGLS, %	16 ± 4	21 ± 2	< 0.001
LVGWI, mmHg %	1576 ± 386	2031 ± 253	< 0.001
LVGWE, %	91 ± 7	96 ± 2	< 0.05
LVGCW, mmHg %	1814 ± 415	2256 ± 246	< 0.001
LVGWW, mmHg%	129 ± 96	85 ± 45	0.09
Diastole function parameters			
E velocity, cm/s	101 ± 24	101 ± 16	0.90
A velocity, cm/s	86 ± 32	92 ± 26	0.51
E/A ratio	1.2(0.9–1.8)	1.1(0.9–1.4)	0.73
E/e’ ratio	9.9(8.8–13.5)	7.1(6.4–7.8)	< 0.001
LAVI, ml/m^2^	29(24–34)	23(21–26)	< 0.05

*FD* Fabry disease, *LVDD* left ventricular end-diastolic diameter, *IVS* interventricular septum, *LVPW* left ventricular posterior wall, *LVMI* left ventricular mass index, *LVEF* left ventricular ejection fractions, *LVGLS* left ventricular global longitudinal strain, *LVGWI* left ventricular global myocardial work index, *LVGWE* left ventricular global myocardial work efficiency, *LVGCW* left ventricular global myocardial constructive work, *LVGWW* left ventricular global myocardial work waste, *LAVI* left atrial volume index.

### Myocardial performance during exercise

**Fig. 3:**
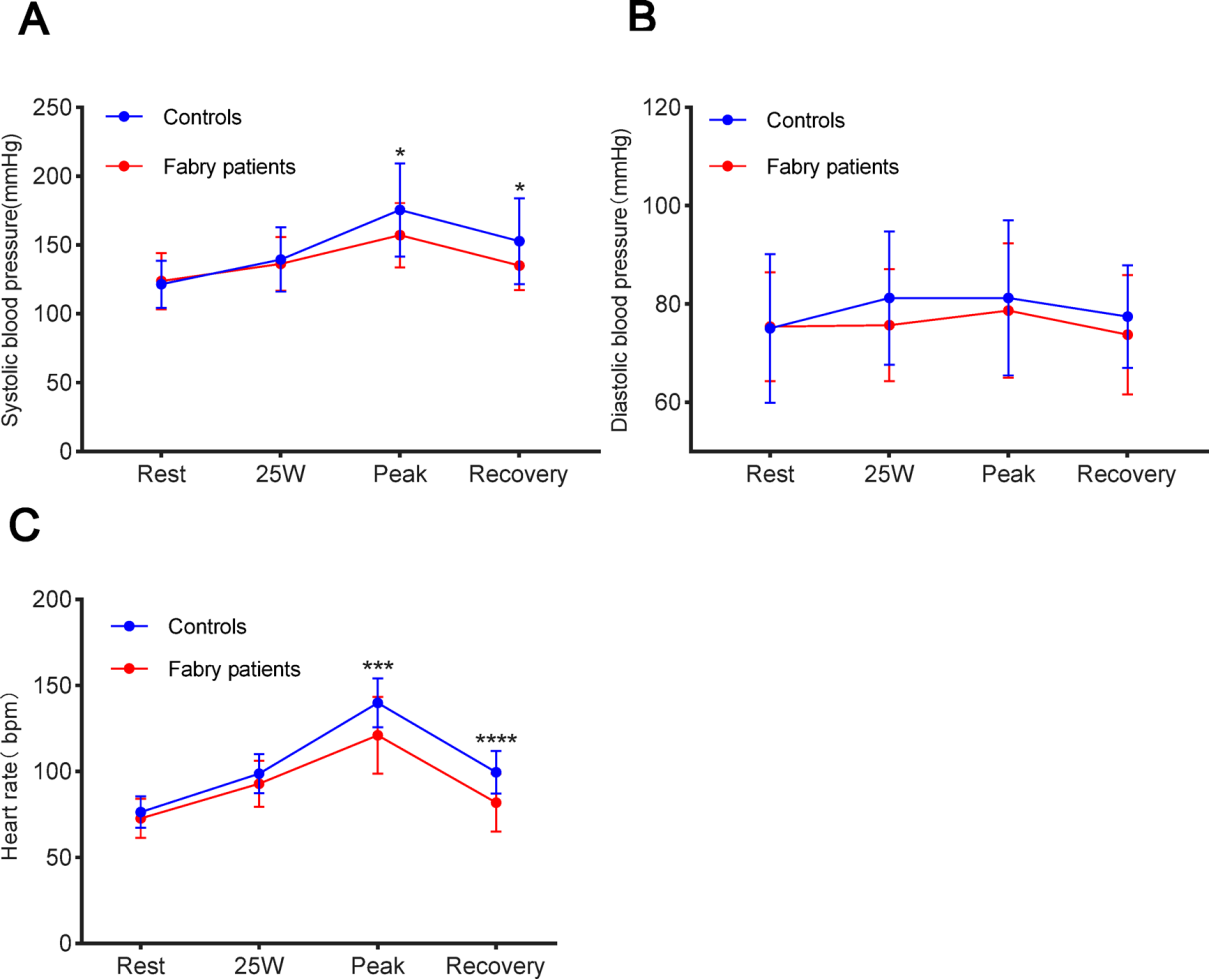
Margin plots with 95% CIs (bars) during exercise in reference to (**A**) SBP, (**B**) DBP, and (**C**) Heart rate in patients with FD and control subjects. *SBP* systolic blood pressure, *DBP* diastolic blood pressure, *FD* Fabry disease. *Significant difference between patients with FD and control subjects (*P* < 0.05).

**Fig. 4:**
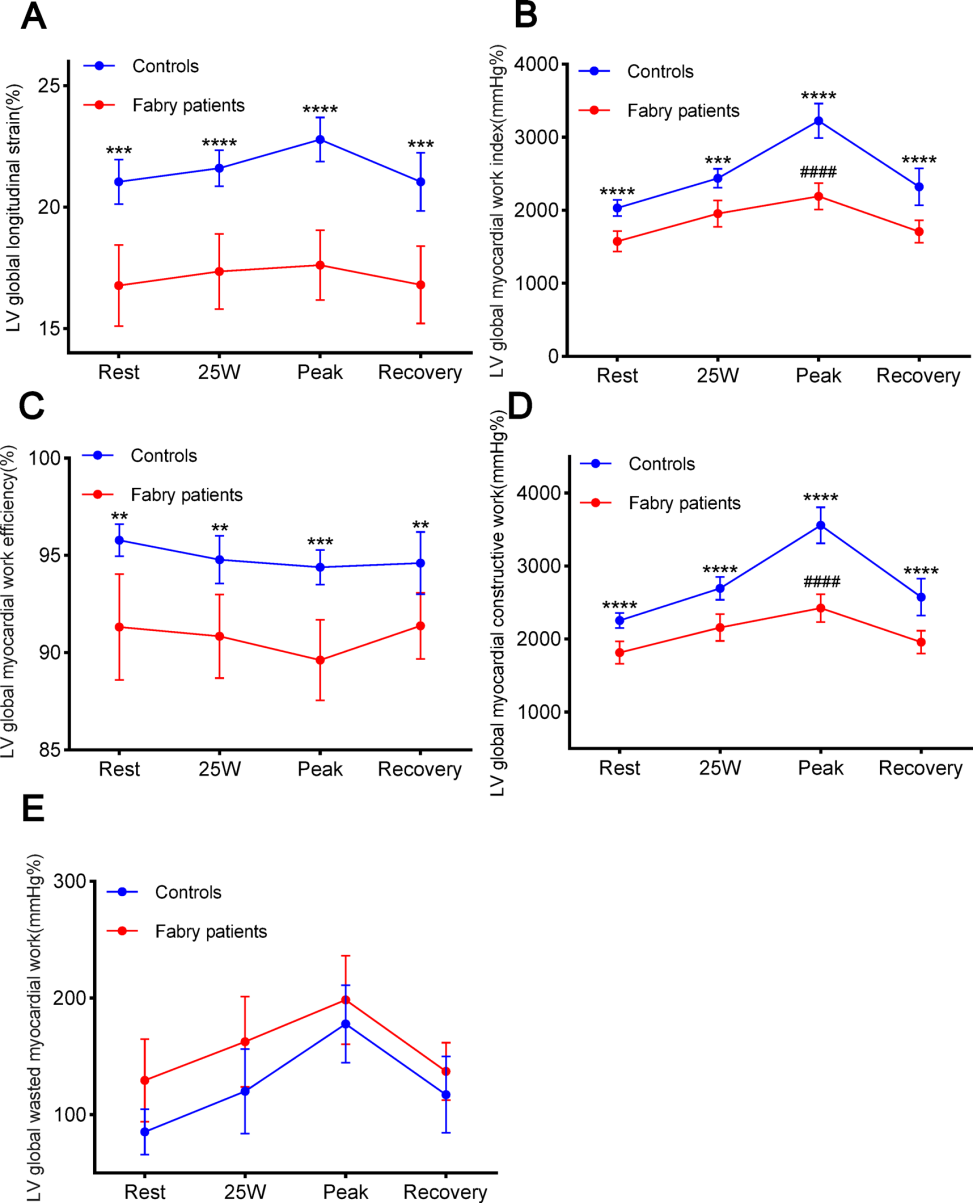
Margin plots with 95% CIs (bars) during exercise in reference to (**A**) LVGLS, (**B**) LVGWI, (**C**) LVGWE, (**D**) LVGCW, and (**E**) LVGWW in patients with FD and control subjects. *LVGLS* left ventricular global longitudinal strain, *LVGWI* left ventricular global myocardial work index, *LVGWE* left ventricular global myocardial work efficiency, *LVGCW* left ventricular global myocardial constructive work, *LVGWW* left ventricular global myocardial work waste. *significant difference between patients with FD and control subjects (*P* < 0.05), # Significant difference in the magnitude of growth from Rest stage to Peak stage between patients with FD and control subjects (*P* < 0.05).

The average exercise duration in FD patients was 5.9 ± 1.6 min, and the average peak workload was 84.8 ± 23.5 W. For controls, the corresponding values were 8.1 ± 1.9 min and 116.3 ± 27.8 W. HR and blood pressure (BP) increased markedly after exercise in both patients with FD and the control cohort. At peak exercise and during the recovery phase, patients with FD had markedly lower HR and SBP relative to the control cohort, though no significant difference was noted in DBP between the two cohorts (rest: *P* = 0.07; 25 W: *P* = 0.10; peak: *P* = 0.46; recovery: *P* = 0.19; Fig. 3). Figure 4 shows the changes in LVGLS and LVMW during exercise. With increasing exercise, LVGLS, LVGWI, LVGCW, and LVGWW markedly increased in both cohorts, while LVGWE decreased. At all phases (rest, 25 W, peak, and recovery), LVGLS, LVGWI, LVGWE, and LVGCW were lower in individuals with FD relative to the control cohort, with no significant differences in LVGWW between the two cohorts (rest: *P* = 0.09; 25 W: *P* = 0.05; peak: *P* = 0.42; recovery: *P* = 0.19). Furthermore, patients with FD showed a markedly smaller elevation in LVMWI and LVGCW from rest to peak exercise relative to the control cohort.

**Fig. 5:**
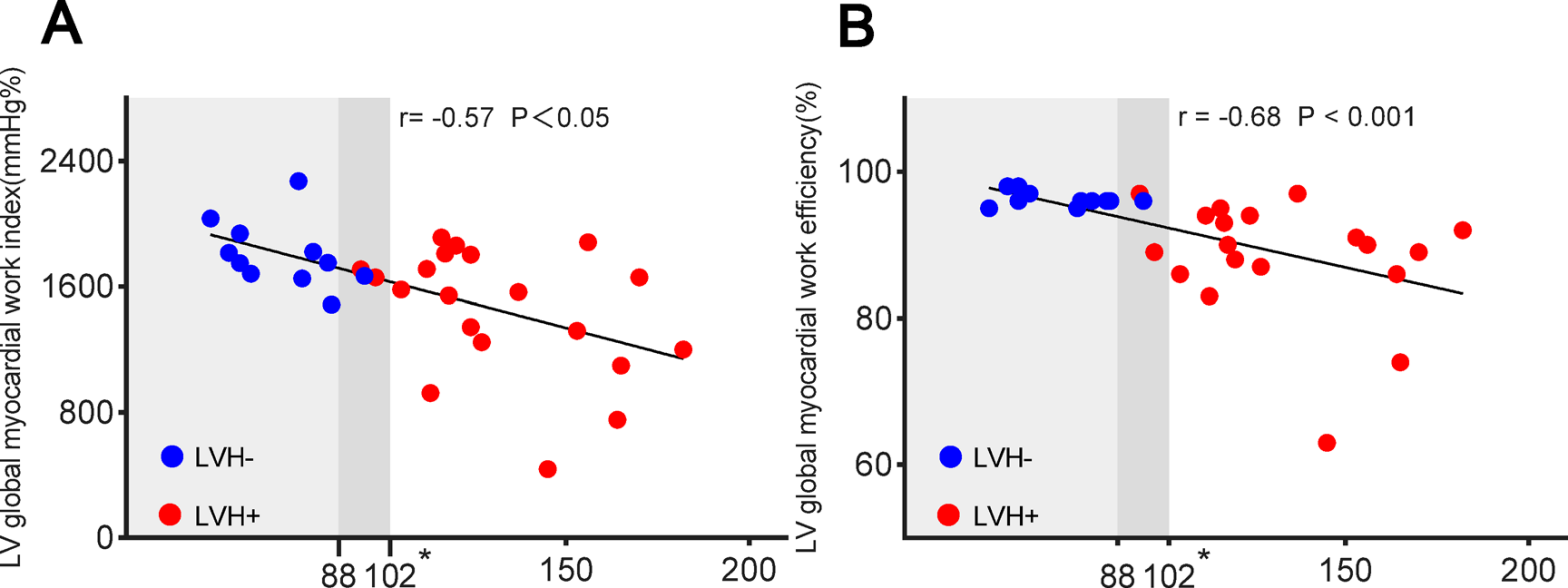
Correlation between (**A**) LVGWI (**B**) LVGWE vs. LVMI. *LVH* left ventricular hypertrophy, *LVGWI* left ventricular global myocardial work index, *LVGWE* left ventricular global myocardial work efficiency, *LVMI* left ventricular mass index. *LVH is defined as an LVMI > 88 g/m^2^ for women or > 102 g/m^2^ for men.

A moderate correlation was observed between LVGWI and LVGWE with LVMI in patients with FD (LVGWI: r = − 0.57, *P* < 0.05; LVGWE: r = − 0.68, *P* < 0.001; Fig. 5). Table 3 compares LVGLS and LVMW at rest and during exercise in two cohorts: individuals with FD without LVH and patients with FD and LVH. From rest to peak exercise, patients with FD with LVH had lower LVGLS, LVGWE, and LVGCW compared to those without LVH.

**Table 3:** LVGLS and MW in FD patients.

	FD patients without LVH (n = 11)	FD patients with LVH (n = 20)	*P* value
LVMI, g/m^2^	73 ± 14	132 ± 25	< 0.001
Resting conditions			
SBP, mmHg	111 ± 7	125 ± 13	< 0.05
DBP, mmHg	75 ± 11	76 ± 11	0.85
LVGLS, %	21 ± 2	15 ± 4	< 0.001
LVGWI, mmHg %	1805 ± 213	1450 ± 406	< 0.05
LVGWE, %	96 ± 1	89 ± 8	< 0.001
LVGCW, mmHg %	2,100 ± 268	1657 ± 401	< 0.05
LVGWW, mmHg%	73 ± 30	160 ± 107	< 0.05
Exercise conditions (Peak stage)			
SBP, mmHg	146 ± 26	161 ± 21	0.10
DBP, mmHg	70 ± 13	83 ± 12	< 0.05
LVGLS, %	21 ± 3	15 ± 3	< 0.001
LVGWI, mmHg %	2,395 ± 451	2081 ± 490	0.09
LVGWE, %	93 ± 4	88 ± 6	< 0.05
LVGCW, mmHg %	2706 ± 500	2273 ± 482	< 0.05
LVGWW, mmHg%	151 ± 75	225 ± 109	0.06

*MW* myocardial constructive work, *FD* Fabry disease, *LVH* left ventricular hypertrophy, *SBP* systolic blood pressure, *DBP* diastolic blood pressure, *LVMI* left ventricular mass index, *LVGLS* left ventricular global longitudinal strain, *LVGWI* left ventricular global myocardial work index, *LVGWE* left ventricular global myocardial work efficiency, *LVGCW* left ventricular global myocardial constructive work, *LVGWW* left ventricular global myocardial work waste.

**Fig. 6:**
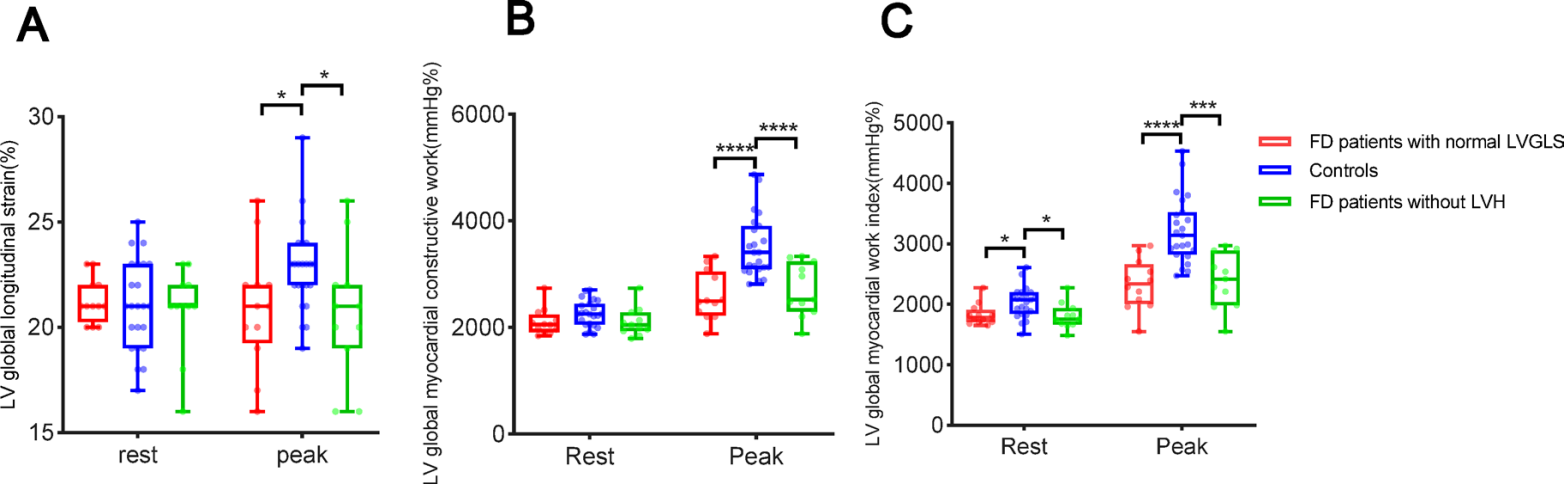
Box Plot at Rest and Peak stage in reference to (**A**) LVGLS, (**B**) LVGCW, and (**C**) LVGWI in patients with FD with normal LVGLS at rest, patients with FD without LHV, and control subjects. *LVH* left ventricular hypertrophy, *LVGLS* left ventricular global longitudinal strain, *LVGCW* left ventricular global myocardial constructive work, *LVGWI* left ventricular global myocardial work index. *significant difference between patients with FD and control subjects (*P* < 0.05).

**Fig. 7:**
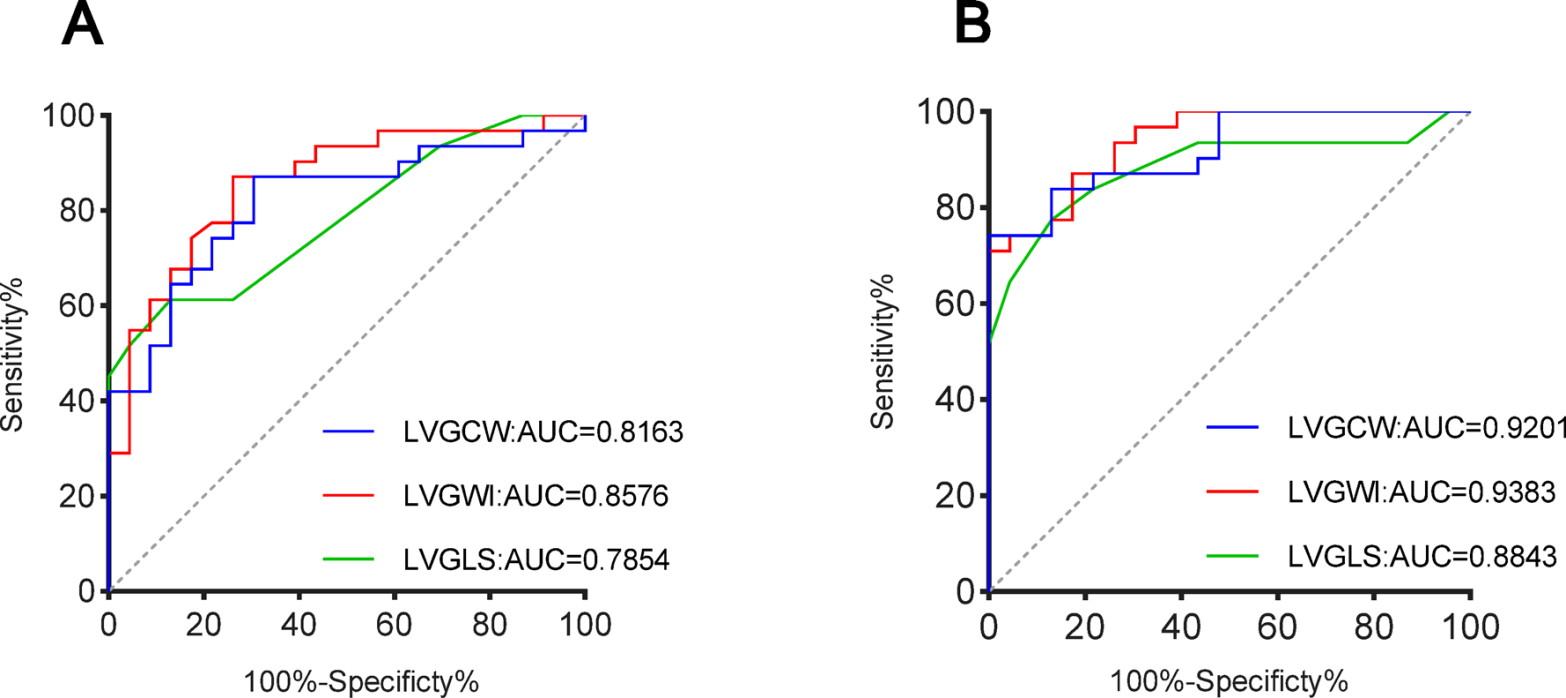
ROC curves for LVMW parameters in comparison to LVGLS at rest and exercise stage to assess LV dysfunction, respectively. (**A**) rest stage, (**B**) exercise stage. *LVGLS* left ventricular global longitudinal strain, *LVGCW* left ventricular global myocardial constructive work, *LVGWI* left ventricular global myocardial work index.

Table 4 reveals that patients with FD with normal LVGLS at rest or those without LVH had markedly lower LVGWI than the control cohort during the resting phase. However, at peak exercise, LVGLS, LVGWI, and LVGCW were dramatically lower in patients with FD (Fig. 6). Figure 7 illustrates the ROC curves of the three parameters LVMWI, LVGCW, and LVGLS for the resting and exercise stages to assess LV dysfunction. In the resting stage, LVGWI (AUC 0.86, sensitivity 87%, specificity 74%) and LVGCW (AUC of 0.82, sensitivity 87%, specificity 70%) diagnostic performance were superior to LVGLS (AUC 0.79, sensitivity 61%, specificity 87%). In the exercise stage, the AUC values of LVGCW, LVGWI, and LVGLS were enhanced, LVGWI (AUC 0.94, sensitivity 71%, specificity 96%) and LVGCW (AUC 0.92, sensitivity 84%, specificity 87%) diagnostic performance remained superior to LVGLS (AUC 0.88, sensitivity of 77%, and specificity of 87%).

**Table 4:** Comparison of LVGLS and LVMW in FD Patients with normal LVGLS, FD Patients without LVH, and Control subjects.

	FD patients without LVH (n = 11)	FD Patients with normal LVGLS at rest (n = 12)	Control subjects (n = 23)	*P* value	*P*^a^ value
Resting conditions					
LVGLS, %	21 ± 2	22 ± 1	21 ± 2	0.71	0.97
LVGWI, mmHg %	1805 ± 213	1828 ± 177	2031 ± 253	< 0.05	< 0.05
LVGWE, %	96 ± 1	93 ± 3	96 ± 2	0.92	0.76
LVGCW, mmHg %	2101 ± 268	2101 ± 249	2256 ± 246	0.08	0.10
LVGWW, mmHg%	73 ± 30	146 ± 73	85 ± 45	0.73	0.58
Exercise conditions (Peak stage)					
LVGLS, %	21 ± 3	21 ± 3	23 ± 2	< 0.05	< 0.05
LVGWI, mmHg %	2395 ± 451	2332 ± 411	3224 ± 547	< 0.001	< 0.001
LVGWE, %	93 ± 4	93 ± 3	94 ± 2	0.25	0.10
LVGCW, mmHg %	2706 ± 500	2594 ± 454	3560 ± 598	< 0.001	< 0.05
LVGWW, mmHg%	151 ± 75	146 ± 73	177 ± 76	0.24	0.34

*FD* Fabry disease, *LVH* left ventricular hypertrophy, *LVGLS* left ventricular global longitudinal strain, *LVGWI* left ventricular global myocardial work index, *LVGWE* left ventricular global myocardial work efficiency, *LVGCW* left ventricular global myocardial constructive work, *LVGWW* left ventricular global myocardial work waste.

^a^Comparison Control subjects to FD without LVH patients.

## Discussion

This study assesses LVMW in patients with FD and the control cohort at rest and during exercise. The key findings of this investigation are as follows: (1) Patients with FD exhibit markedly reduced LVGWI, LVGWE, and LVGCW, with these reductions being more pronounced during exercise compared to healthy controls; (2) The reserves of LVGWI and LVGCW are markedly reduced in patients with FD than healthy controls; (3) The presence of LVH indicates more severe impairment of LVMW in patients with FD; and (4) LVMW combined with Stress Echocardiography allows early detection of impaired cardiac function in Fabry patients.

In this study, patients with FD had lower BSA and BMI compared to the control cohort, likely due to intracellular lipid accumulation, which disrupts normal energy metabolism and fat storage^17^. When FD affects the digestive system, it can result in malabsorption, anorexia, and gastrointestinal symptoms such as diarrhea or constipation^18^. The elevated E/e’ ratio observed in patients with FD reflects increased LV diastolic pressure, suggesting impaired diastolic function. This dysfunction is attributed to the accumulation of Lyso-Gb-3 and Gb-3 in cardiac cells and tissues, which leads to markedly reduced myocardial elasticity and compliance^19^.

Previous research reported that approximately 46% of FD patients experienced a drop in DBP during exercise, suggesting impaired sympathetic vasoconstriction^20^. Abnormal cardiovascular responses to exercise, particularly involving blood pressure and heart rate regulation, have been associated with autonomic dysfunction in FD^21^. In our study, while FD patients exhibited lower peak heart rate and systolic blood pressure compared to controls, no significant diastolic pressure drop was observed during exercise or recovery. Several factors may account for this discrepancy, including differences in exercise modality (semi-supine cycling vs. upright treadmill), disease stage, and reduced exercise tolerance^21^. In addition, the attenuated heart rate response observed in patients compared to controls may suggest the presence of chronotropic incompetence. These findings warrant further investigation into autonomic regulation in FD and its relationship to exercise capacity and cardiovascular risk.

Prior research has demonstrated that LVGLS is essential for the early diagnosis of FD^22^ and in the prognostic evaluation of affected patients^23^. Additionally, a markedly reduced longitudinal strain in the basal portions of the LV is a distinctive feature of FD, differentiating it from other conditions associated with LVH^24^. Compared to earlier studies, our research focused on the disparities in LVMW between individuals with FD and the control cohort.

The non-invasive myocardial work assessed with the LV pressure-strain loop is a novel echocardiographic parameter that combines strain with afterload, providing a more comprehensive evaluation of myocardial function. While it is unlikely to replace LVGLS, this parameter may complement existing strain assessments, enhancing their diagnostic value^25^. Unlike LVGLS, which only measures peak systolic strain, LVMW evaluates the entire cardiac cycle, allowing for a more holistic evaluation of both systolic and diastolic function. This makes LVMW a more accurate indicator of overall cardiac performance and a valuable indicator of the myocardium’s mechanical performance and metabolic state. Additionally, LVMW can quantify myocardial energy consumption, offering valuable insights into myocardial metabolism and energy utilization efficiency^26^. LVMW has been widely adopted in the evaluation of cardiac resynchronization therapy, hypertension, HCM, acute coronary syndromes, aortic stenosis, myocardial amyloidosis, and chemotherapy-related myocardial injury^27^.

In the present study, patients with FD exhibited markedly reduced LVGWI, LVGWE, and LVGCW relative to the control cohort. This reduction may be linked to the accumulation of Lyso-Gb-3 and Gb-3 in myocardial cells, leading to cell death. Furthermore, the accumulation of these substances in vascular endothelial cells can cause myocardial ischemia, which may induce myocardial fibrosis and subsequently reduce myocardial contractility^2^. Kristopher et al.^28^ have reported decreased myocardial perfusion in patients with FD, particularly in the subendocardial regions, with more severe regional fibrosis. Furthermore, Steudel et al.^29^ demonstrated in their investigation of CA and FD that layered radial strain, particularly endocardial radial strain, enhances diagnostic accuracy. During exercise, LVGWI, LVGWE, and LVGCW are worse in patients with FD than in the control cohort. This deterioration could result from a mismatch between myocardial supply and demand during exercise. Conditions such as LVH or microvascular disease may reduce coronary blood flow reserve, trigger ischemia, and impair myocardial contractility^13^. These issues can be detected through echocardiography, which reveals LV dysfunction^12^. This underscores the overall compromised myocardial function in patients with FD, who exhibit poor exercise capacity and reduced myocardial work efficiency.

In the control cohort, myocardial reserve augments cardiac deformation during physical activity, with LVGLS typically increasing by about 5.3% from rest to peak exercise^30^. However, the elevation in cardiac afterload during exercise can partially offset this improvement in myocardial deformation. Since LVGWI and LVGCW incorporate afterload conditions, they may offer additional value over LVGLS in evaluating cardiac function under exercise conditions. Our findings suggest that while there is no substantial variance in the increase of LVGLS from rest to peak exercise between the control cohort and individuals with FD, there are significant differences in LVGWI and LVGCW. Fan et al.^15^ established that LVGWI and LVGCW surpass conventional measures like GLS by mitigating load dependence and more precisely quantifying myocardial energy efficiency. These parameters also showed superior diagnostic accuracy in distinguishing infiltrative cardiomyopathies (e.g., FD and AL-CA) from hypertensive heart disease.

In our study, LVGWI and LVGWE in patients with FD were moderately correlated with the LVMI. Patients with FD who had left LVH exhibited markedly lower LVGWI, LVGCW, and LVGWE at rest than those without LVH. Research has shown that the gradual buildup of glycosphingolipids in the myocardial cells of individuals with FD leads to LV wall thickening^31^. As the disease progresses, irreversible fibrosis develops, reflecting irreversible cardiac damage associated with LVH^32^. Additionally, studies have indicated that patients with FD with severe LVH were more likely to experience adverse cardiovascular events^33^. LVGLS deteriorates with the progression of LVH in patients with FD^22^. Since LVMW is derived from LVGLS, it can provide a more comprehensive assessment of cardiac function when combined with LVGLS. The calculation of LVMW is relatively easy, requiring only the measurement of LVGLS, blood pressure, and the timing of aortic and mitral valve events. According to current guidelines, regardless of symptoms, preadult (< 18 years of age) men with classic FD should be treated with enzyme replacement therapy (ERT), which is usually initiated before cardiac involvement is detected to prevent potential cardiac damage, while cardiac reserve capacity can be assessed using LVMW and stress echocardiography^34,35^. However, non-classic FD generally does not have extracardiac danger signals, and diagnostic delays have not improved significantly in recent years, so early detection of impairment of cardiac function using LVMW and stress echocardiography is is crucial for timely initiation of disease-specific therapies, including enzyme replacement or chaperone therapy, which are known to improve myocardial mass and function when initiated before irreversible fibrosis^36^. The study demonstrated that enzyme replacement therapy enhances exercise capacity in the first year, which may correlate with reverse myocardial changes^37^. The early recognition of cardiac function may also influence follow-up intervals and therapeutic decisions. For example, FD patients displaying normal resting but impaired stress LVMW might benefit from more frequent monitoring (every 6 months rather than annually) and early consideration of treatment escalation—even in the absence of LV hypertrophy or fibrosis. Additionally, such patients may be considered for clinical trials evaluating early interventions aimed at preserving myocardial work performance.

Finally, from a prognostic perspective, although myocardial fibrosis detected by late enhancement on CMR is a well-established predictor of adverse outcomes^38^, stress echocardiography offers a noninvasive, widely available, low-cost alternative to detect early functional impairment^39^. Serial assessment of stress LVMW could guide individualized treatment strategies, optimize timing of therapy initiation, and potentially improve long-term cardiac outcomes in FD.

### Limitations

Given the constraints of the relatively limited case cohort in this investigation, the statistical power may be insufficient to represent the entire FD population fully. This study utilized stress echocardiography, and during exercise, increased HR may compromise the accuracy of capturing end-systolic and end-diastolic images, potentially affecting the assessment of myocardial deformation. Additionally, changes in patient position and increased respiration rate may impact ultrasound image quality, thereby influencing the accuracy and reliability of the results. LVMW is derived from LVGLS analysis, and its accuracy is highly dependent on the precision of the measurements of LVGLS. If the quality and frame rate of 2D images are not sufficiently high, speckle tracking may fail to accurately localize in each frame, leading to errors in the measurements of LVGLS. Accumulated errors in LVGLS will ultimately affect the numerical evaluation of LVMW, reducing its accuracy. Furthermore, errors may arise when the device automatically identifies mitral and aortic valve opening and closing times. Manual intervention may be required, making the operator’s experience a potential factor influencing the results.

This study employed myocardial work analysis primarily focused on global cardiac function assessment. Although segmental analysis may help identify regional functional abnormalities, the current global evaluation strategy is sufficient to meet the main objectives of this study, considering that FD typically presents with diffuse myocardial involvement. Future research could incorporate imaging modalities with higher spatial resolution, such as cardiac magnetic resonance imaging, to conduct more detailed regional functional analyses. In addition, all enrolled patients were newly diagnosed with FD and had not yet received enzyme replacement therapy (ERT) or cardiac-targeted therapies. Since key biochemical markers reflecting end-organ damage (including estimated glomerular filtration rate, high-sensitivity troponin T, and N-terminal pro-B-type natriuretic peptide) were not systematically analyzed, future studies should incorporate these parameters and further evaluate the effects of standard therapies on myocardial work and cardiac function to provide a more comprehensive assessment of treatment outcomes in FD patients.

## Conclusion

Patients with FD exhibit markedly lower LVGWI, LVGWE, and LVGCW compared to the healthy controls, with these differences becoming more pronounced during exercise. Although LVGWI and LVGCW increase in patients with FD during exercise, the rate of increase is reduced, suggesting impaired myocardial metabolism and energy utilization efficiency. This impairment is more severe in patients with FD and LVH. Additionally, LVMW combined with Stress Echocardiography allows early detection of impaired cardiac function in Fabry patients.

## Data Availability

The datasets generated during and analyzed during the current study are available from the corresponding author on reasonable request.
